# Physical activity and checkpoint inhibition: association with toxicity and survival

**DOI:** 10.1093/jnci/djad245

**Published:** 2023-11-24

**Authors:** Rik J Verheijden, Anna Cabané Ballester, Karel C Smit, Mick J M van Eijs, Cheryl P Bruijnen, Anne S R van Lindert, Karijn P M Suijkerbuijk, Anne M May

**Affiliations:** Julius Center for Health Sciences and Primary Care, University Medical Center Utrecht, Utrecht University, Utrecht, the Netherlands; Department of Medical Oncology, University Medical Center Utrecht, Utrecht University, Utrecht, the Netherlands; Julius Center for Health Sciences and Primary Care, University Medical Center Utrecht, Utrecht University, Utrecht, the Netherlands; Julius Center for Health Sciences and Primary Care, University Medical Center Utrecht, Utrecht University, Utrecht, the Netherlands; Department of Medical Oncology, University Medical Center Utrecht, Utrecht University, Utrecht, the Netherlands; Department of Medical Oncology, University Medical Center Utrecht, Utrecht University, Utrecht, the Netherlands; Center for Translational Immunology, University Medical Center Utrecht, Utrecht University, Utrecht, the Netherlands; Department of Medical Oncology, University Medical Center Utrecht, Utrecht University, Utrecht, the Netherlands; Department of Pulmonology, University Medical Center Utrecht, Utrecht University, Utrecht, the Netherlands; Department of Medical Oncology, University Medical Center Utrecht, Utrecht University, Utrecht, the Netherlands; Julius Center for Health Sciences and Primary Care, University Medical Center Utrecht, Utrecht University, Utrecht, the Netherlands

## Abstract

**Background:**

Although animal experiments suggest beneficial effects of physical activity (PA) on antitumor immunity, little is known about the effects of PA on immune checkpoint inhibitor (ICI) toxicity and effectiveness in humans. We assessed the association of PA with immune-related adverse events (irAE) and survival in patients undergoing ICI.

**Methods:**

Patients receiving ICI who completed the Dutch short questionnaire to assess health enhancing physical activity (SQUASH) questionnaire at the start of treatment as part of the prospective UNICIT study in an academic hospital were included. PA was quantified by calculating total metabolic equivalent task hours per week (total PA) and hours per week of moderate- to vigorous-intensity PA during sport and leisure time (MVPA-SL). Associations of PA with severe irAE occurrence within 1 year and overall survival (OS) were evaluated using logistic regression and Cox proportional hazard regression, respectively, with adjustment for probable confounders.

**Results:**

In total, 251 patients were included, with a median follow-up of 20 months. Moderate and high levels of total PA were associated with lower odds of severe irAE occurrence compared to low levels of total PA (adjusted OR: 0.34 [95% CI = 0.12 to 0.90] and 0.19 [95% CI = 0.05 to 0.55], respectively). Moderate and high levels of total PA were also associated with prolonged survival (adjusted HR: 0.58 [95% CI = 0.32 to 1.04] and 0.48 [95% CI = 0.27 to 0.89], respectively). Similar associations were observed in patients who performed more MVPA-SL.

**Conclusions:**

Higher physical activity levels at the start of ICI treatment are associated with lower risk of severe irAEs and probably prolonged survival. Randomized controlled trials are needed to investigate whether patients indeed benefit from increasing PA levels after diagnosis.

Over the past decade, the emergence of immune checkpoint inhibitors (ICI) has tremendously extended survival for many individuals with cancer (1-3). Despite the clinical benefits, ICI can cause immune-related adverse events (irAEs). These irAEs can affect almost every organ system, and they range from mild to severe with some cases being fatal (4,5). The incidence and onset of irAEs can vary between patients and the type of ICIs administered, typically occurring within the first 4 months after therapy initiation (6). Only 5% to 7% of patients experience irAEs more than 1 year after ICI initiation (7,8). irAEs may cause considerable morbidity and, if severe, often require ICI discontinuation and prompt immunosuppression. Nevertheless, irAE occurrence has been associated with prolonged survival (9,10).

Several studies have linked higher levels of physical activity (PA) among patients treated with chemotherapy to improved overall survival and diminished side effects (11,12). As the mechanism of action of ICI is entirely different from that of chemotherapy, reflected by differential patterns of response and resistance and a distinct toxicity profile, it is unknown whether the advantageous effects of PA in patients receiving chemotherapy also apply to patients receiving ICI. In animal models, exercise was associated with increased cytotoxic (CD8^+^) T cell and natural killer (NK) cell activity, and decreased regulatory T cells and myeloid derived suppressor cells (MDSCs) (13-19), suggesting beneficial effects on antitumor immunity. Furthermore, animal studies have demonstrated that exercise combined with ICI led to more reduction in tumor growth rate than ICI alone (13,14,16,17,20). In humans, PA has been correlated with reduced risk of developing conventional autoimmune diseases including rheumatoid arthritis and Crohn disease (18,21,22). However, the association of PA with ICI-induced immune-related toxicity and ICI efficacy has not yet been studied.

Here, we aimed to investigate the association of PA at the start of ICI treatment with the occurrence of severe irAEs and survival.

## Methods

### Study sample

Patients starting with ICI in the University Medical Center Utrecht, a Dutch academic cancer center, are prospectively enrolled in the ongoing UNICIT biobank (23). Biospecimens and clinical data are collected before and early during ICI treatment, and health-related questionnaires are filled in before ICI initiation. Patients are followed up and clinical data are recorded periodically. For this study, adult patients undergoing ICI treatment with anti-programmed cell death (ligand) 1 (anti-PD-[L]1) monotherapy, combined ICI (cICI) with anti-cytotoxic T-lymphocyte-associated protein 4 (anti-CTLA-4) plus anti-PD-1, or anti-PD-1 plus chemotherapy (ICI + chemotherapy) between October 2019 and October 2022 were included and followed up until January 31, 2023. The UNICIT biobank study was not considered subject to the Dutch Medical Research with Human Subjects Law by the medical research ethics committee and was approved by the institutional biobank review committee (Tcbio 18-123). All participants provided written informed consent.

### Assessment of physical activity

Physical activity was assessed at treatment initiation using the validated Dutch short questionnaire to assess health enhancing physical activity (SQUASH; Supplementary Material, available online) (24). The aim of the SQUASH is to estimate the intensity and amount of PA during an average week in the past few months. The questions were categorized into the following topics: commuting activities, activities at work and school, household tasks, and sports and leisure time activities. The self-reported values were transformed into a metabolic equivalent of task (MET) score for each activity and domain, based on the Ainsworth compendium of physical activities categorization (25). The levels of intensity were ranked as light-intensity (<3.0 METs), moderate-intensity (3.0 to 5.9 METs), or vigorous-intensity (≥6.0 METs) (26). Total physical activity, quantified in MET-hours, was calculated as the sum of the time per week spent on each activity multiplied by their corresponding MET value. Moderate- to vigorous-intensity physical activity during sports and leisure time (MVPA-SL) was quantified as time (in hours) spent on activities with ≥3.0 MET within sports and leisure time domains, and includes leisure time bicycling, gardening, odd jobs, and up to 4 different sports (11).

### Outcome assessment

IrAEs were reported by the treating physician according to the Common Terminology Criteria for Adverse Events (CTCAE), version 5 (27). Since most irAEs occur within the first year (7), severe irAEs (defined as grade 3 or higher) from first-line ICI within the first year after ICI initiation were considered during logistic regression to minimize the impact of death as a competing risk. Only patients who started ICI at least 1 year before data cutoff were considered for analyses on irAEs to prevent misclassification. Overall survival (OS) was defined as the time from ICI initiation until death. Patients were censored at last follow-up date if they were still alive at that time.

### Statistical analysis

Since no well-established cutoffs for PA exist, total PA was split into 3 categories based on tertiles. MVPA-SL was categorized as no time spent on MVPA-SL, and for the remainder of patients in up to median (6 hours/week) or above median. These categories were consistently used throughout all analyses. To explore the possible correlation between PA and occurrence of severe irAEs within 1 year from ICI initiation, we conducted multivariable logistic regression. Since we anticipated a nonlinear relationship between PA and severe irAE occurrence, we fitted PA as restricted cubic splines with 3 knots to allow for flexibility. The adjusted odds ratio (OR_adj_) with 95% confidence interval (CI) of each value of PA relative to zero was visualized. Additionally, to account for death as a competing risk of severe irAEs, a Fine and Gray subdistribution hazard model was applied.

To assess the possible association between PA and OS, Kaplan-Meier curves were presented. Multivariable Cox proportional hazard regression was used to estimate adjusted hazard ratios (HR_adj_) with 95% CI. The proportional hazards assumption was not violated according to visual inspection of Schoenfeld residuals. As for toxicity analyses, we anticipated a nonlinear relationship between PA and OS (as confirmed by Martingale residuals) and fitted PA as restricted cubic splines.

All models were adjusted for age, sex, type of primary tumor, treatment setting (unresectable or metastatic vs adjuvant or curative), prior systemic treatment, and ICI therapy type. All patients were included in multivariable analyses, despite a sensitivity analysis with additional adjustment for Eastern Cooperative Oncology Group (ECOG) performance status (28), out of which 6 patients were excluded because performance status was not reported. All statistical analyses were conducted using R, version 4.3.1, with a 2-sided alpha of .05 considered significant.

## Results

### Patient population

In total, 251 patients were included in this study, with a median follow-up duration of 20 months. The majority of patients were male (66.5%), the mean age was 64 years (SD 12.4), and most patients received treatment for unresectable or metastatic cancer (59.8%; Table 1). Patients reported a median of 75.7 MET-hours per week (range: 0 to 370.6 MET-hours/week) and spent a median of 4.0 hours per week on MVPA-SL (range 0 to 38.5 hours/week). Whereas 70.2% of patients belonging to the most active group according to total PA (highest tertile) had melanoma, this was only 46.7% in the most inactive group (lowest tertile).

**Table 1.** Patient characteristics by tertiles of total physical activity per week^a^

	Low PA	Moderate PA	High PA	Overall
	[0-51]	(51-101]	(101-371]	[0-371]
	(n = 84)	(n = 83)	(n = 84)	(n = 251)
Total PA (MET-hours/week)				
median [Q1-Q3]	30.0 [16.9-39.9]	75.7 [62.3-86.4]	128.9 [112.7-165.6]	75.7 [40.0-112.7]
MVPA-SL (hours/week)				
median [Q1-Q3]	0.9 [0-2.4]	5.0 [1.5-8.0]	8.9 [4.4-15.0]	4.0 [0.9-8.5]
Sex				
male	54 (64.3%)	60 (72.3%)	53 (63.1%)	167 (66.5%)
female	30 (35.7%)	23 (27.7%)	31 (36.9%)	84 (33.5%)
Age (years)				
mean (SD)	64.7 (12.5)	62.7 (13.2)	61.2 (11.2)	62.9 (12.4)
[min-max]	[26.0-85.0]	[20.0-73.0]	[37.0-94.0]	[20.0-91.0]
ECOG performance status				
0	31 (36.9%)	39 (47.0%)	56 (66.7%)	126 (50.2%)
1	38 (45.2%)	41 (49.4%)	22 (26.2%)	101 (40.2%)
2	12 (14.3%)	1 (1.2%)	3 (3.6%)	16 (6.4%)
3	2 (2.4%)	0 (0.0%)	0 (0.0%)	2 (0.8%)
unknown	1 (1.2%)	2 (2.4%)	3 (3.6%)	6 (2.4%)
Tumor type				
melanoma	40 (47.6%)	54 (65.1%)	59 (70.2%)	153 (61.0%)
NSCLC	18 (21.4%)	8 (9.6%)	12 (14.3%)	38 (15.1%)
RCC	10 (11.9%)	9 (10.8%)	9 (10.7%)	28 (11.2%)
Other	16 (19.0%)	12 (14.5%)	4 (4.8%)	32 (12.7%)
Stage				
III	25 (29.8%)	38 (45.8%)	41 (48.8%)	104 (41.4%)
IV	58 (69.0%)	43 (51.8%)	43 (51.2%)	144 (57.4%)
other	1 (1.2%)	2 (2.4%)	0 (0.0%)	3 (1.2%)
Treatment setting				
unresectable or metastatic	62 (73.8%)	46 (55.4%)	42 (50.0%)	150 (59.8%)
adjuvant or curative	22 (26.2%)	37 (44.6%)	42 (50.0%)	101 (40.2%)
Previous systemic treatment				
none	61 (72.6%)	73 (88.0%)	72 (85.7%)	206 (82.1%)
ICI	2 (2.4%)	2 (2.4%)	2 (2.4%)	6 (2.4%)
chemo/targeted therapy	21 (25.0%)	8 (9.6%)	10 (11.9%)	39 (15.5%)
Therapy				
anti-PD-(L)1	52 (61.9%)	60 (72.3%)	53 (63.1%)	165 (65.7%)
cICI	21 (25.0%)	18 (21.7%)	22 (26.2%)	61 (24.3%)
ICI + chemo or targeted therapy	11 (13.1%)	5 (6.0%)	9 (10.7%)	25 (10.0%)

a Low PA, moderate PA, and high PA refer to tertiles of total weekly MET-hours. PA = physical activity; MET = metabolic equivalent task; MVPA-SL = moderate- to vigorous-intensity physical activity during sports and leisure time; Q1 = first quartile, Q3 = third quartile; n = number of patients; ECOG = Eastern Cooperative Oncology Group; ICI = immune checkpoint inhibition; anti-PD-(L)1 = anti-programmed cell death (ligand) 1 monotherapy; cICI = combination immune checkpoint inhibition (anti-CTLA4+anti-PD1); NSCLC = non-small-cell lung carcinoma; RCC = renal cell carcinoma.

### Physical activity and severe immune-related adverse events

A total of 209 patients started at least 1 year before data cutoff and were thus included in irAE analyses. Thirty-eight of these patients developed a severe irAEs within the first year. One patient developed the first severe irAE after 16 months, which is after the 1-year landmark, and was thus classified as “without severe irAE” in logistic regression analyses. Types of severe irAEs per ICI regimen are shown in Supplementary Table 1 (available online).

Among 73 patients with low levels of total PA, 21 (29%) had severe irAEs within 1 year, compared with 10 out of 69 (14%) patients with moderate levels of total PA and 7 out of 67 (10%) patients with high levels of MET-hours (Table 2). Patients reporting moderate or high levels of total PA had lower odds of developing severe irAEs within 1 year than patients with the lowest levels of total PA (OR_adj_: 0.34; 95% CI = 0.12 to 0.90 and 0.19; 95% CI = 0.05 to 0.55, respectively). This was supported by flexibly modeling the adjusted odds ratio of severe irAEs for each possible unit of total PA compared to zero MET-hours/week (*P* = .012; Supplementary Figure 1, A, available online), with a steep decline in the odds until approximately 100 MET-hours per week, after which the OR stabilized or attenuated and uncertainty increased. Similarly, low and high levels of MVPA-SL were associated with lower odds of developing irAEs compared to no MVPA-SL at all (OR_adj_: 0.25; 95% CI = 0.09 to 0.69 and 0.16; 95% CI = 0.05 to 0.47, respectively). This was in line with the visualized flexible model (*P* = .004; Supplementary Figure 1, B, available online), although there were too few patients with high MVPA-SL to draw conclusions on (non)linearity.

**Table 2.** Incidence of severe immune-related adverse events within 1 year according to physical activity^a^

	Total n	Incidence 1 y (%)	OR_crude_ (95% CI)	OR_adj, excl-ECOG_ (95% CI)	OR_adj, ECOG_ (95% CI)
Total PA (MET-hours/week)				*P* = .012^a^	*P* = .012^a^
Low	73	21 (29%)	–	–	–
Moderate	69	10 (14%)	0.42 (0.17 to 0.95)	0.34 (0.12 to 0.90)	0.32 (0.10 to 0.88)
High	67	7 (10%)	0.29 (0.11 to 0.70)	0.19 (0.05-0.55)	0.20 (0.06 to 0.60)
MVPA-SL (hours/week)				*P* = .004^a^	*P* = .004^a^
Zero	47	17 (36%)	–	–	–
Low	80	12 (15%)	0.31 (0.13 to 0.73)	0.25 (0.09 to 0.69)	0.22 (0.07 to 0.62)
High	82	9 (11%)	0.22 (0.08 to 0.53)	0.16 (0.05 to 0.47)	0.13 (0.04 to 0.42)

a Likelihood ratio test *P* value for multivariable model with PA using restricted cubic splines versus model without PA. PA = physical activity; MVPA-SL = moderate- to vigorous-intensity physical activity during sports and leisure time in hours per week; MET = metabolic equivalent task; OR_crude_ = unadjusted odds ratio; OR_adj_ = odds ratio adjusted for sex, age, tumor type, setting, previous systemic therapy, type of therapy, and in case of OR_adj, ECOG_ Eastern Cooperative Oncology Group performance status.

Results were similar when additionally adjusting for ECOG performance status (Table 2; Supplementary Figure 2, available online). When restricting to cICI-treated patients, similar but nonsignificant associations between higher PA at cICI initiation and lower odds of severe irAE occurrence were observed (Supplementary Table 2; Supplementary Figure 3, available online). To further account for death as a competing risk of severe irAEs, Fine and Gray subdistribution hazard regression was conducted in all 251 patients, which confirmed previous observations (Supplementary Table 3; Supplementary Figure 4, available online).

### Physical activity and overall survival

**Figure 1.**
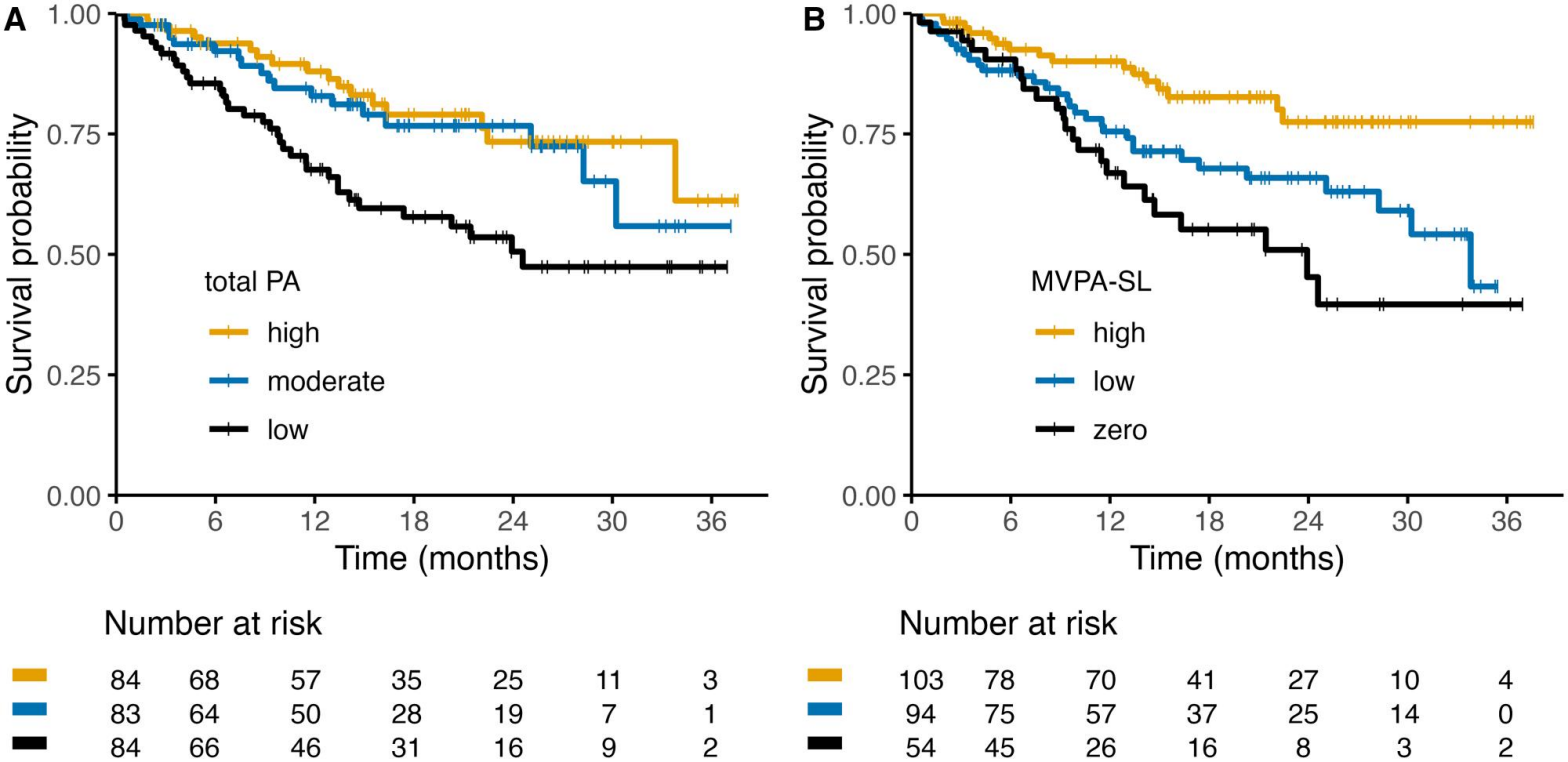
Association between physical activity at immune checkpoint inhibitor initiation and overall survival. Kaplan-Meier curves (**A, B**) of overall survival of patients with cancer treated with immune checkpoint inhibitors stratified by physical activity levels. **A**) Metabolic equivalent task (MET) hours per week. **B**) Time spent on moderate- to vigorous-intensity activities during sports and leisure time (MVPA-SL).

Among 251 patients, a total of 70 deaths occurred during follow-up, and median overall survival was not reached. The probability to be alive 1 year after ICI initiation was 0.68 (95% CI = 0.58 to 0.79) for patients with low levels of total PA and 0.83 (95% CI = 0.74 to 0.92) and 0.88 (95% CI = 0.81 to 0.96) for patients with moderate and high levels of MET-hours, respectively (Table 3; Figure 1, A). Moderate and high levels of total PA were associated with improved OS, compared with the lowest tertile (HR_adj_: 0.58; 95% CI = 0.32 to 1.04 and 0.48; 95% CI = 0.27 to 0.89, respectively). Compared with zero MET-hours per week, there was a steep decline of the hazard of death until approximately 100 MET-hours per week, after which the hazard attenuated slightly and uncertainty increased (*P* = .019; Supplementary Figure 5, A, available online). This is suggestive of a plateau after approximately 100 MET-hours per week, in line with the survival curves in Figure 1, A.

**Table 3.** Overall survival according to physical activity^a^

	Total n	Incidence 1 y (1 y survival probability)	HR_crude_ (95% CI)	HR_adj, excl-ECOG_ (95% CI)	HR_adj, ECOG_ (95% CI)
Total PA (MET-hours/week)				*P* = .019^a^	*P* = .279^a^
Low	84	25 (0.68)	–	–	–
Moderate	83	12 (0.83)	0.51 (0.29 to 0.90)	0.58 (0.32 to 1.04)	0.69 (0.37 to 1.29)
High	84	9 (0.88)	0.42 (0.24 to 0.76)	0.48 (0.27 to 0.89)	0.60 (0.32 to 1.11)
MVPA-SL (hours/week)				*P* = .036^a^	*P* = .192^a^
Zero	54	25 (0.68)	–	–	–
Low	94	12 (0.83)	0.51 (0.29 to 0.90)	0.58 (0.32 to 1.04)	0.73 (0.40 to 1.34)
High	103	9 (0.88)	0.42 (0.24 to 0.76)	0.48 (0.27 to 0.89)	0.48 (0.24 to 0.96)

a Likelihood ratio test *P* value for multivariable model with PA using restricted cubic splines versus model without PA. PA = physical activity; MVPA-SL = moderate- to vigorous-intensity physical activity during sports and leisure time in hours per week; MET = metabolic equivalent task; HR_crude_ = unadjusted hazard ratio; HR_adj_ = odds ratio adjusted for sex, age, tumor type, setting, previous systemic therapy, type of therapy, and in case of HR_adj, ECOG_ Eastern Cooperative Oncology Group performance status.

A similar nonlinear association was observed between MVPA-SL and OS, although less strong (*P* = .036). There was a nonsignificant trend toward prolonged survival in patients with low or high levels of MVPA-SL compared with no MVPA-SL (HR_adj_: 0.58; 95% CI = 0.32 to 1.04) and 0.48; 95% CI = 0.27 to 0.89), respectively; Figure 1, B). Similarly, there seemed to be a plateau after approximately 10 hours per week spend on MVPA-SL (Supplementary Figure 5, B, available online), but uncertainty is large.

Similar associations between PA and OS were observed in the subgroup of patients with unresectable tumors (thus excluding adjuvant ICI; Supplementary Table 4; Supplementary Figure 6, available online). When restricting the analyses to melanoma patients with the addition of lactate dehydrogenase (LDH) as a possible confounder, when restricting to patients with nonmelanoma tumors, and when restricting to patients with ECOG performance status 0 or 1, comparable trends toward prolonged survival with higher PA levels were observed, although no longer significant (Supplementary Tables 5-7; Supplementary Figures 7-9, available online, respectively). When additionally adjusting for ECOG performance status among all patients, associations of PA with OS were less strong and no longer significant (Table 3; Supplementary Figure 10, available online).

## Discussion

In this prospective observational study, we observed that moderate to high PA at the start of ICI treatment is associated with reduced risk of severe irAEs and probably with prolonged OS. These associations were found for both overall PA (measured as total MET-hours/week) and weekly time spent on moderate- to vigorous-intensity PA during sport and leisure time.

In chemotherapy treated patients, PA has been associated with better survival and decreased side effects (11,12). However, in ICI-treated patients, data on the possible benefits of PA were lacking. We observed a reduced risk of severe irAEs in more active patients. This is in line with observations that higher PA is associated with reduced risk of developing conventional autoimmune diseases (21,22). Furthermore, a rat model of ulcerative colitis demonstrated that swimming attenuated dextran sulfate sodium-induced chronic colitis, as reflected by reduction in colon shortening, fecal calprotectin levels, activated CD3^+^ T cell abundance within the lamina propria, and proinflammatory serum cytokine levels including tumor necrosis factor (TNF), interleukin (IL)-1β and IL-6, with an increase in serum levels of IL-10, an immunosuppressive cytokine (29). Interestingly, we observed that PA was independently associated with severe irAEs, whereas ECOG performance status was not. This is in line with our previous study of 819 anti-PD-1-treated patients with advanced melanoma, which did not demonstrate an association between ECOG performance status and severe irAEs (30). Performance status is a measure of patients’ level of functioning, including their ability to care for themselves, their daily activity, and their physical ability (31). Although patients with ECOG performance status 2 to 4 are likely inactive, PA may vary largely among patients with performance status 0 or 1. Questionnaire-based PA measurements may more accurately reflect the PA a patient actually conducted and may hence be more closely correlated to physiological differences.

We observed prolonged OS in more active patients, even after adjusting for possible confounders. Recently, Liu et al. reported on a retrospective study in which they asked 59 patients or an immediate family member with unresectable hepatocellular carcinoma who started combined Lenvatinib plus anti-PD-1 therapy 3 months to 2.5 years before the phone call about their PA levels before or within 1 month after therapy initiation (17). Similar to our findings, they concluded that regular PA (based on the American College of Sports Medicine and American Heart Association Physical Activity Recommendations) (32) improved combined Lenvatinib plus anti-PD-1 efficacy. Recall bias may have influenced the results of this study, since patients with good outcomes may overreport their PA levels, whereas family members of already deceased patients may be more conservative.

Preclinical studies have demonstrated that exercise may slow tumor growth upon ICI treatment (13,14,16,17,20). This beneficial effect of exercise may be the result of intratumoral blood vessel normalization (13), increased CD8^+^ T cell infiltration and cytotoxicity (13,14), increased intratumoral NK cell activity (15), reduced intratumoral regulatory T cell abundance (16,17), and reduced intratumoral MDSC abundance and immunosuppressive function (14,16), although results are conflicting as extensively reviewed elsewhere (19,33-35). The exact mechanisms by which PA would reduce autoimmunity while enhancing anti-tumor immunity are not yet well understood. Given the accumulating evidence of improved tumor response and survival in patients who developed irAEs (10,36), irAEs are hypothesized to reflect the successful reinvigoration of immunity by ICI (37). More research is needed to understand whether PA improves survival while reducing irAE frequency in an immune-related manner.

When additionally adjusting for performance status and when restricting to patients with ECOG performance status 0 or 1, the association of PA with OS was less strong and no longer significant, which was not observed for the association between PA and severe irAEs. This was not surprising, given that a worse performance status is known to be prognostic of poor survival. The association between PA and OS observed in our study may reflect general patient fitness or nonspecific advantageous effects of PA, rather than cancer-specific effects. Although preclinical studies have demonstrated that exercise may hamper tumor growth, and PA seems to have some added predictive effect over performance status in our study, no definite conclusion on the advantageous effects of PA on tumor control can be drawn based on our observational data.

Different measures of PA are used throughout the literature, which is a result of the variety of questionnaires used that do not all cover similar domains of PA (38). Using the SQUASH questionnaire, we analyzed both total physical activity (which includes commuting activities, activities at work or school, household tasks, and sports and leisure time activities) and time spent on moderate- to vigorous-intensity PA during sports and leisure time, both of which were of a similar magnitude as in previous reports (11,39). Patients with moderate and high levels of PA had equally reduced risk of severe irAEs and prolonged survival compared with low levels of PA, with a plateau at approximately 100 MET-hours of total physical activity per week or 10 hours per week spent on MVPA-SL. Although these results should be interpreted with caution given the limited number of patients who are highly active, they are in line with previous observations linking PA with mortality in patients with cancer (40-42), and they suggest that PA is beneficial to a certain extent. The most benefit can be expected from activating inactive patients, whereas stimulating patients who are already active might have less added benefit.

It is important to acknowledge that our study has some limitations. First, the sample size is limited, and the cohort is heterogeneous, warranting further investigation in larger cohort studies. Furthermore, we have assessed only clinician-assessed severe irAEs, but (chronic) low-grade irAEs can be very burdensome. Low-grade irAEs were not documented reliably in our study but should be considered in future research—for example, reported by patients using apps or a diary. Although we used a validated questionnaire, self-reported measures of PA are vulnerable to reporting bias. However, given the prospective design, selective misreporting is not influenced by future severe irAE occurrence or shorter OS. Furthermore, we do not anticipate differences among patients with respect to access to care, since the mandatory insurance system in the Netherlands ensures reimbursement for all citizens. This study was partially conducted during the COVID-19 pandemic, during which the everyday lives and routines of many were disrupted, signifying that the reported PA levels may not accurately reflect all participants’ average week. There is considerable heterogeneity in our cohort with respect to tumor type and ICI regimen, limiting the sample size in subgroup analyses. However, we thoroughly adjusted for possible confounders, we found similar associations with different measures of PA, and trends pointed in the same direction among all subgroups and with different statistical techniques.

Despite the use of a prospective cohort, a validated questionnaire, and reliable reporting of clinical data, no conclusions on causation can be drawn. Given the observational design of our study, residual confounding cannot be completely ruled out. PA likely enhances immune system resilience, but the exact mechanisms by which physical activity may mitigate autoimmunity are not yet fully understood (18). irAEs resemble conventional autoimmune diseases, although differences exist in onset, severity, and pathogenesis. With the opportunity to collect data before the onset of inflammation, irAEs provide a unique opportunity to study how exercise impacts autoimmunity mechanistically. Nevertheless, randomized controlled trials (RCTs) are warranted to explore whether promoting exercise upon cancer diagnosis reduces risk of irAEs and improves OS. Several RCTs investigating the feasibility of exercise interventions during ICI are ongoing, 4 of which additionally assess tumor outcomes (NCT03171064; NCT04263467 (43); NCT04676009 (44); NCT04866810; NCT05358938; ACTRN12619000952145) (45). Adverse events are the secondary endpoint of 1 RCT (NCT04676009) (44) and 1 single-arm exercise intervention study (NCT05103722). Although RCTs are ongoing, and given that physical activity is generally considered safe, our results highlight the importance of a physically active lifestyle in patients with cancer.

In conclusion, higher physical activity levels at the start of ICI treatment are associated with reduced occurrence of severe irAEs and probably with prolonged survival. Future research is needed to validate these findings and to investigate whether patients with low PA levels indeed benefit from increasing PA levels after cancer diagnosis.

## Supplementary Material

djad245_Supplementary_Data

## Data Availability

The data underlying this article cannot be shared due to privacy regulations. Not all patients consented to make their data publicly available. All analysis scripts are available via https://github.com/rjverheijden/UNICIT_ICI_physical_activity.
