# Supplementary material for: Physical activity and checkpoint inhibition: association with toxicity and survival
Source: J Natl Cancer Inst. 2023 Nov 24;116(4):573–9. doi: 10.1093/jnci/djad245 (PMC10995850; doi:10.1093/jnci/djad245)
Supplement: djad245_Supplementary_Data [file djad245_supplementary_data.pdf]

SUPPLEMENTARY MATERIAL

Physical activity and checkpoint inhibition: association with toxicity and survival

Rik J Verheijden, Anna Cabané Ballester, Karel C Smit, Mick JM van Eijs, Cheryl P Bruijnen, Anne SR van Lindert, Karijn PM Suijkerbuijk, Anne M May

- The short questionnaire to assess health enhancing physical activity (SQUASH)
- Supplementary Table 1: Frequencies of types of severe immune-related adverse events per ICI regimen.
- Supplementary Table 2: Association of physical activity at initiation of ipiliumumab+nivolumab with severe immune-related adverse events (irAEs) within one year.
- Supplementary Table 3: Fine and Gray subdistribution hazard model of the association of physical activity at immune checkpoint inhibitor initiation with severe immune-related adverse events (irAE) in patients with cancer, accounting for death as competing risk.
- Supplementary Table 4: Association of physical activity at immune checkpoint inhibitor initiation with overall survival in patients with metastatic or unresectable tumors.
- Supplementary Table 5: Association of physical activity at immune checkpoint inhibitor initiation with overall survival in patients with melanoma.
- Supplementary Table 6: Association of physical activity at immune checkpoint inhibitor initiation with overall survival in patients with nonmelanoma tumors.
- Supplementary Table 7: Association of physical activity at immune checkpoint inhibitor initiation with overall survival in cancer patients with ECOG performance status 0 or 1.
- Supplementary Figure 1: Association between physical activity at immune checkpoint inhibitor initiation and severe immune-related adverse events (irAE) within one year in patients with cancer.
- Supplementary Figure 2: Association between physical activity at immune checkpoint inhibitor initiation and severe immune-related adverse events (irAE) in patients with cancer with additional adjustment for ECOG performance status.
- Supplementary Figure 3: Association between physical activity at immune checkpoint inhibitor initiation and severe immune-related adverse events (irAE) in patients with cancer treated with ipilimumab+nivolumab.
- Supplementary Figure 4: Cumulative incidence functions of severe immune-related adverse events (SirAE) and death according to physical activity at immune checkpoint inhibitor initiation.
- Supplementary Figure 5: Association between physical activity at immune checkpoint inhibitor initiation and overall survival.
- Supplementary Figure 6: Association between physical activity at immune checkpoint inhibitor initiation and overall survival in patients with metastatic or unresectable disease.
- Supplementary Figure 7: Association between physical activity at immune checkpoint inhibitor initiation and overall survival in patients with melanoma.

- Supplementary Figure 8: Association between physical activity at immune checkpoint inhibitor initiation and overall survival in patients with nonmelanoma tumors.
- Supplementary Figure 9: Association between physical activity at immune checkpoint inhibitor initiation and overall survival in patients with cancer with ECOG performance status 0 or 1.
- Supplementary Figure 10: Association between physical activity at immune checkpoint inhibitor initiation and overall survival (OS) in patients with cancer with additional adjustment for ECOG performance status.

The short questionnaire to assess health enhancing physical activity (SQUASH)

Think about an average week in the past months. Please indicate **how many days per week** you performed the following activities, how much time **on average** you were engaged in this, and (if applicable) how strenuous this activity was for you?

**COMMUTING ACTIVITIES (round trip)**	Days per *week*	Average time per *day*	Effort (circle please)
Walking to/from work or school	☐ days	☐ hour ☐☐ minutes	Slow/moderate/fast
Bicycling to/from work or school	☐ days	☐ hour ☐☐ minutes	Slow/moderate/fast
Not applicable	☐		

**ACTIVITY AT WORK AND SCHOOL**	Average time per *week*
Light work<br>(sitting/standing with some walking, e.g., a desk job)	☐ hour ☐☐ minutes
Intense work<br>(walking job, regularly lifting heavy objects at work)	☐ hour ☐☐ minutes

**HOUSEHOLD ACTIVITIES**	Days per *week*	Average time per *day*
Light household work<br>(standing work such as cooking, washing dishes, ironing, child care, and walking work such as vacuuming, doing groceries)	☐ days	☐ hour ☐☐ minutes
Intense household work<br>(scrubbing floor, carpet beating, walking with heavy shopping bags)	☐ days	☐ hour ☐☐ minutes

**LEISURE TIME ACTIVITIES**	Days per *week*	Average time per *day*	Effort (circle please)
Walking	☐ days	☐ hour ☐☐ minutes	Slow/moderate/fast
Bicycling	☐ days	☐ hour ☐☐ minutes	Slow/moderate/fast
Gardening	☐ days	☐ hour ☐☐ minutes	Light/moderate/intense
Odd jobs	☐ days	☐ hour ☐☐ minutes	Light/moderate/intense
Sports (Please write down yourself)<br>*e.g., tennis, fitness, skating, swimming, dancing*			
1…………………………………………….	☐ days	☐ hour ☐☐ minutes	Light/moderate/intense
2…………………………………………….	☐ days	☐ hour ☐☐ minutes	Light/moderate/intense
3…………………………………………….	☐ days	☐ hour ☐☐ minutes	Light/moderate/intense
4…………………………………………….	☐ days	☐ hour ☐☐ minutes	Light/moderate/intense

Supplementary Table 1: Frequencies of types of severe immune-related adverse events per ICI regimen.

Type of gr≥3 irAE	ipilimumab+nivolumab (N=61)	anti-PD-(L)1 ± chemo/targeted therapy (N=190)
colitis	4 (13.8%)	6 (33.3%)
dermatitis	2 (6.9%)	0 (0.0%)
diabetes mellitus type 1	2 (6.9%)	0 (0.0%)
duodenitis	1 (3.4%)	1 (5.6%)
cholangitis	0 (0.0%)	1 (5.6%)
encephalitis	0 (0.0%)	1 (5.6%)
gastritis	3 (10.3%)	0 (0.0%)
hepatitis	5 (17.2%)	1 (5.6%)
hyperthyroidism	1 (3.4%)	0 (0.0%)
meningitis	2 (6.9%)	0 (0.0%)
myocarditis	2 (6.9%)	0 (0.0%)
myositis	1 (3.4%)	1 (5.6%)
pancreatitis	0 (0.0%)	3 (16.7%)
pericarditis	0 (0.0%)	1 (5.6%)
pneumonitis	4 (13.8%)	3 (16.7%)
polyneuropathy	1 (3.4%)	0 (0.0%)
sarcoid like reaction	1 (3.4%)	0 (0.0%)
**total**	29 (47.5%)	18 (9.5%)

Supplementary Table 2: Association of physical activity at initiation of ipiliumumab+nivolumab with severe immune-related adverse events (irAEs) within one year.

**Total physical activity (MET-hours/week)**	**OR_{adj} (95%CI) of severe irAE**
Low [0 to 51]	ref
Intermediate (51 to 101]	0.46 (0.10-1.89)
High (101 to 371]	0.54 (0.97-1.06)
**Moderate to vigorous leisure time and sports (hours/week)**	**OR_{adj} (95%CI) of severe irAE**
Zero [0]	ref
Low (0 to 6]	0.37 (0.08-1.56)
High (6 to 38.5]	0.41 (0.08-1.98)

Abbreviations: OR: odds ratio; CI: confidence interval; MET: Metabolic Equivalent Task. These analyses are adjusted for sex and age.

Supplementary Table 3: Fine and Gray subdistribution hazard model of the association of physical activity at immune checkpoint inhibitor initiation with severe immune-related adverse events (irAE) in patients with cancer, accounting for death as competing risk.

**Total physical activity (MET-hours/week)**	**HR (95%CI) of severe irAE**
Low [0 to 51]	ref
Intermediate (51 to 101]	0.47 (0.21-1.05)
High (101 to 371]	0.43 (0.21-0.90)
**Moderate to vigorous leisure time and sports (hours/week)**	**HR (95%CI) of severe irAE**
Zero [0]	ref
Low (0 to 6]	0.30 (0.13-0.66)
High (6 to 38.5]	0.37 (0.18-0.78)

HR, subdistribution hazard ratio; CI confidence interval; MET, Metabolic equivalent task. These analyses are adjusted for: sex, age, tumor type, setting (unresectable/metastatic vs adjuvant/curative), previous systemic therapy, and type of therapy.

Supplementary Table 4: Association of physical activity at immune checkpoint inhibitor initiation with overall survival in patients with metastatic or unresectable tumors.

**Total physical activity (MET-hours/week)**	**HR_{adj} (95%CI) of death**
Low [0 to 51]	ref
Intermediate (51 to 101]	0.55 (0.29-1.04)
High (101 to 371]	0.39 (0.19-0.81)
**Moderate to vigorous leisure time and sports (hours/week)**	**HR_{adj} (95%CI) of death**
Zero [0]	ref
Low (0 to 6]	0.79 (0.44-1.42)
High (6 to 38.5]	0.27 (0.12-0.62)

Abbreviations: HR: hazard ratio; CI: confidence interval; MET: Metabolic Equivalent Task. These analyses are adjusted for sex, age, previous systemic therapy, and type of therapy.

Supplementary Table 5: Association of physical activity at immune checkpoint inhibitor initiation with overall survival in patients with melanoma.

**Total physical activity (MET-hours/week)**	**HR_{adj} (95%CI) of death**
Low [0 to 51]	ref
Intermediate (51 to 101]	0.79 (0.31-2.03)
High (101 to 371]	0.56 (0.21-1.50)
**Moderate to vigorous leisure time and sports (hours/week)**	**HR_{adj} (95%CI) of death**
Zero [0]	ref
Low (0 to 6]	0.17 (0.06-0.51)
High (6 to 38.5]	0.20 (0.07-0.57)

Abbreviations: HR: hazard ratio; CI: confidence interval; MET: Metabolic Equivalent Task. These analyses are adjusted for sex, age, setting (unresectable/metastatic vs adjuvant/curative), previous systemic therapy, type of therapy, and LDH levels.

Supplementary Table 6: Association of physical activity at immune checkpoint inhibitor initiation with overall survival in patients with nonmelanoma tumors.

**Total physical activity (MET-hours/week)**	**HR_{adj} (95%CI) of death**
Low [0 to 51]	ref
Intermediate (51 to 101]	0.48 (0.22-1.05)
High (101 to 371]	0.47 (0.21-1.06)
**Moderate to vigorous leisure time and sports (hours/week)**	**HR_{adj} (95%CI) of death**
Zero [0]	ref
Low (0 to 6]	1.25 (0.62-2.52)
High (6 to 38.5]	0.52 (0.21-1.29)

Abbreviations: HR: hazard ratio; CI: confidence interval; MET: Metabolic Equivalent Task. These analyses are adjusted for sex, age, setting (unresectable/metastatic vs adjuvant/curative), previous systemic therapy, and type of therapy.

Supplementary Table 7: Association of physical activity at immune checkpoint inhibitor initiation with overall survival in cancer patients with ECOG performance status 0 or 1.

**Total physical activity (MET-hours/week)**	**HR_{adj} (95%CI) of death**
Low [0 to 51]	ref
Intermediate (51 to 101]	0.74 (0.39-1.38)
High (101 to 371]	0.61 (0.32-1.15)
**Moderate to vigorous leisure time and sports (hours/week)**	**HR_{adj} (95%CI) of death**
Zero [0]	ref
Low (0 to 6]	0.64 (0.34-1.19)
High (6 to 38.5]	0.45 (0.23-0.92)

Abbreviations: HR: hazard ratio; CI: confidence interval; MET: Metabolic Equivalent Task. These analyses are adjusted for sex, age, tumor type, setting (unresectable/metastatic vs adjuvant/curable), and type of therapy.

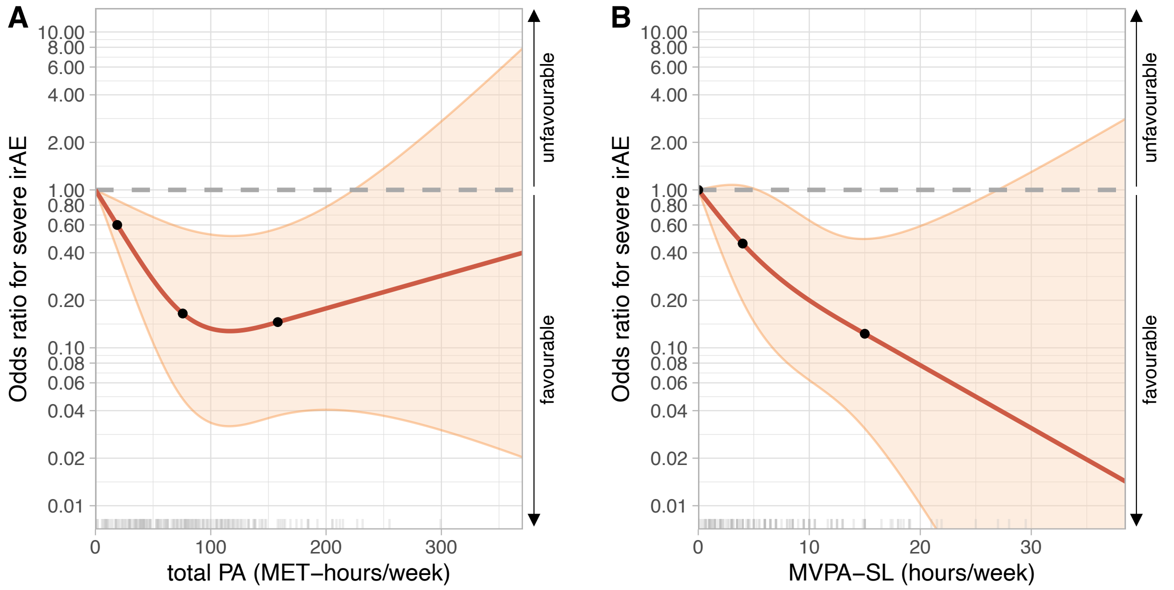

Supplementary Figure 1: Association between physical activity at immune checkpoint inhibitor initiation and severe immune-related adverse events (irAE) within one year in patients with cancer. a Metabolic Equivalent Task (MET) hours per week. **b** Time spent on moderate-to-vigorous-intensity physical activity during sports and leisure time (MVPA-SL). These curves represent odds ratio (OR) with 95% confidence interval (CI) with 0 as reference and are adjusted for sex, age, tumor type, treatment setting, previous systemic therapy, and type of therapy.

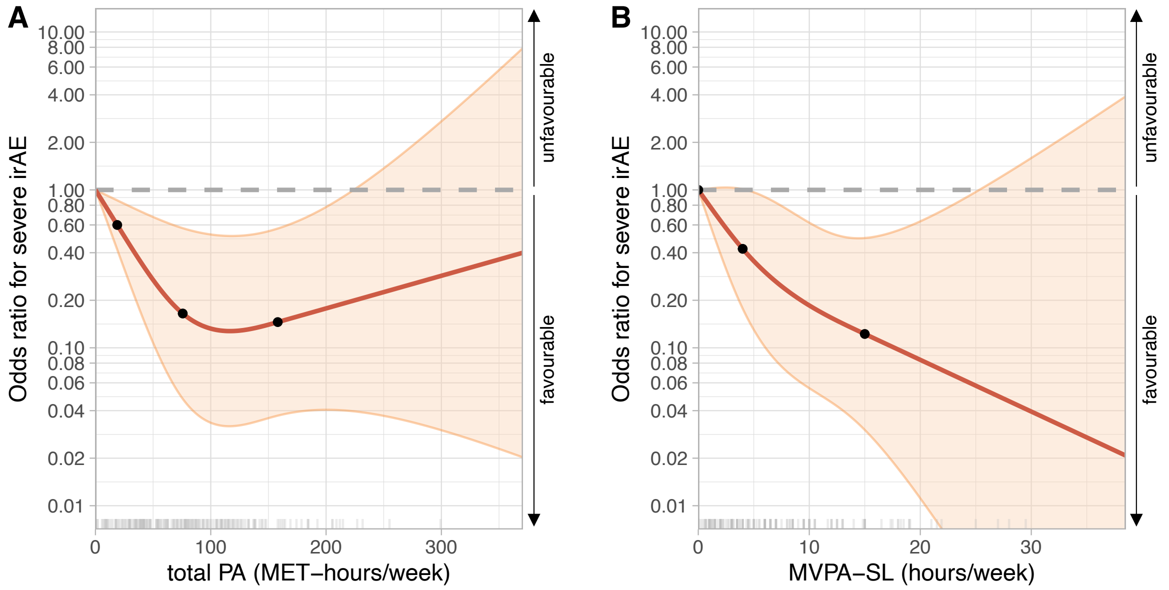

Supplementary Figure 2: Association between physical activity at immune checkpoint inhibitor initiation and severe immune-related adverse events (irAE) in patients with cancer with additional adjustment for ECOG performance status. a Metabolic Equivalent Task (MET) hours per week. **b** Time spent on moderate-to-vigorous-intensity physical activity during sports and leisure time (MVPA-SL). These curves represent odds ratio (OR) with 95% confidence interval (CI) with 0 as reference and are adjusted for sex, age, ECOG performance status, tumor type, treatment setting, previous systemic therapy, and type of therapy.

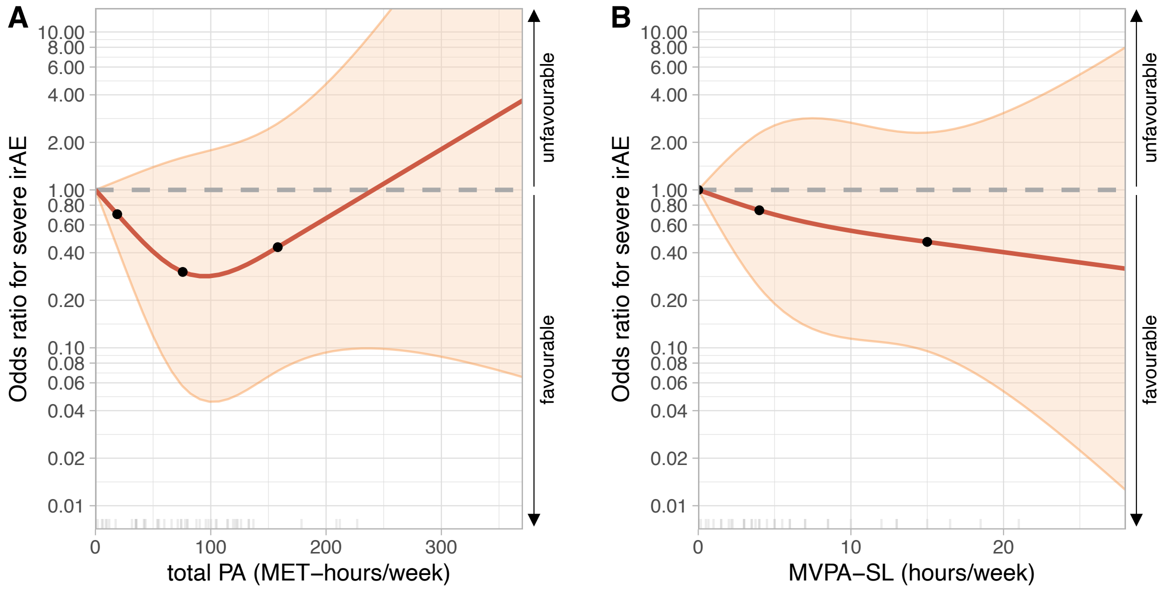

Supplementary Figure 3: Association between physical activity at immune checkpoint inhibitor initiation and severe immune-related adverse events (irAE) in patients with cancer treated with ipilimumab+nivolumab. a Metabolic Equivalent Task (MET) hours per week. **b** Time spent on moderate-to-vigorous-intensity physical activity during sports and leisure time (MVPA-SL). These curves represent odds ratio (OR) with 95% confidence interval (CI) with 0 as reference and are adjusted for sex, age, tumor type, treatment setting and previous systemic therapy.

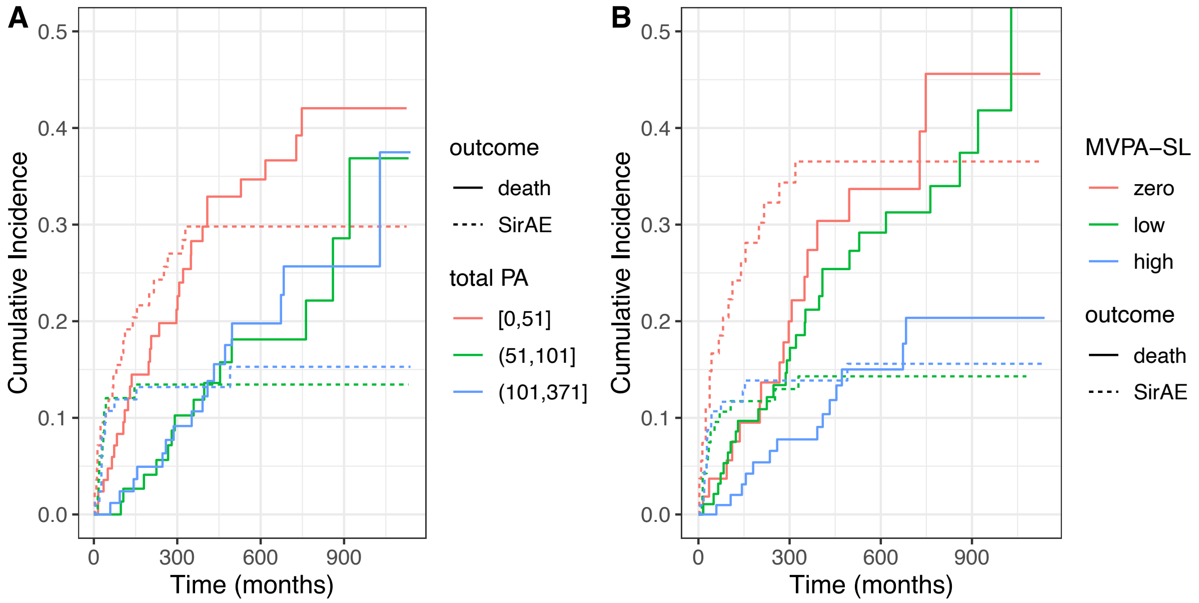

Supplementary Figure 4: Cumulative incidence functions of severe immune-related adverse events (SirAE) and death according to physical activity at immune checkpoint inhibitor initiation. a Metabolic Equivalent Time (MET) hours per week; **b** time spent on moderate-to-vigorous-intensity physical activity during sports and leisure time (MVPA-SL).

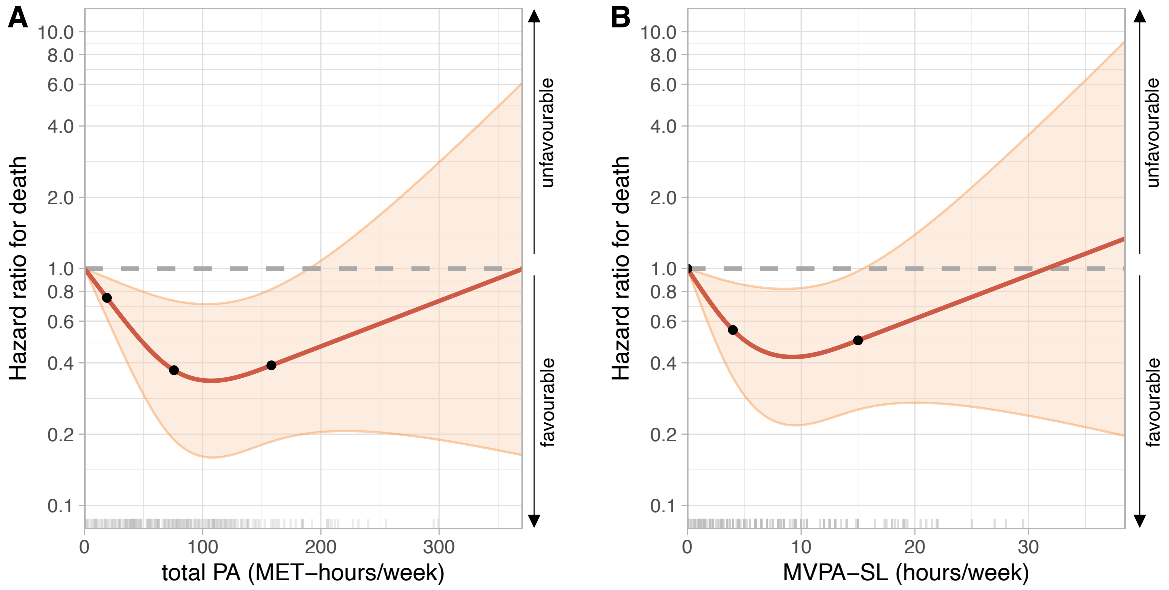

Supplementary Figure 5: Association between physical activity at immune checkpoint inhibitor initiation and overall survival. a Metabolic Equivalent Task (MET) hours per week. **b** Time spent on moderate-to-vigorous-intensity physical activity during sports and leisure time (MVPA-SL). These curves represent hazard ratio (HR) with 95% confidence interval (CI) with 0 as reference and are adjusted for sex, age, tumor type, treatment setting previous systemic therapy, and type of therapy.

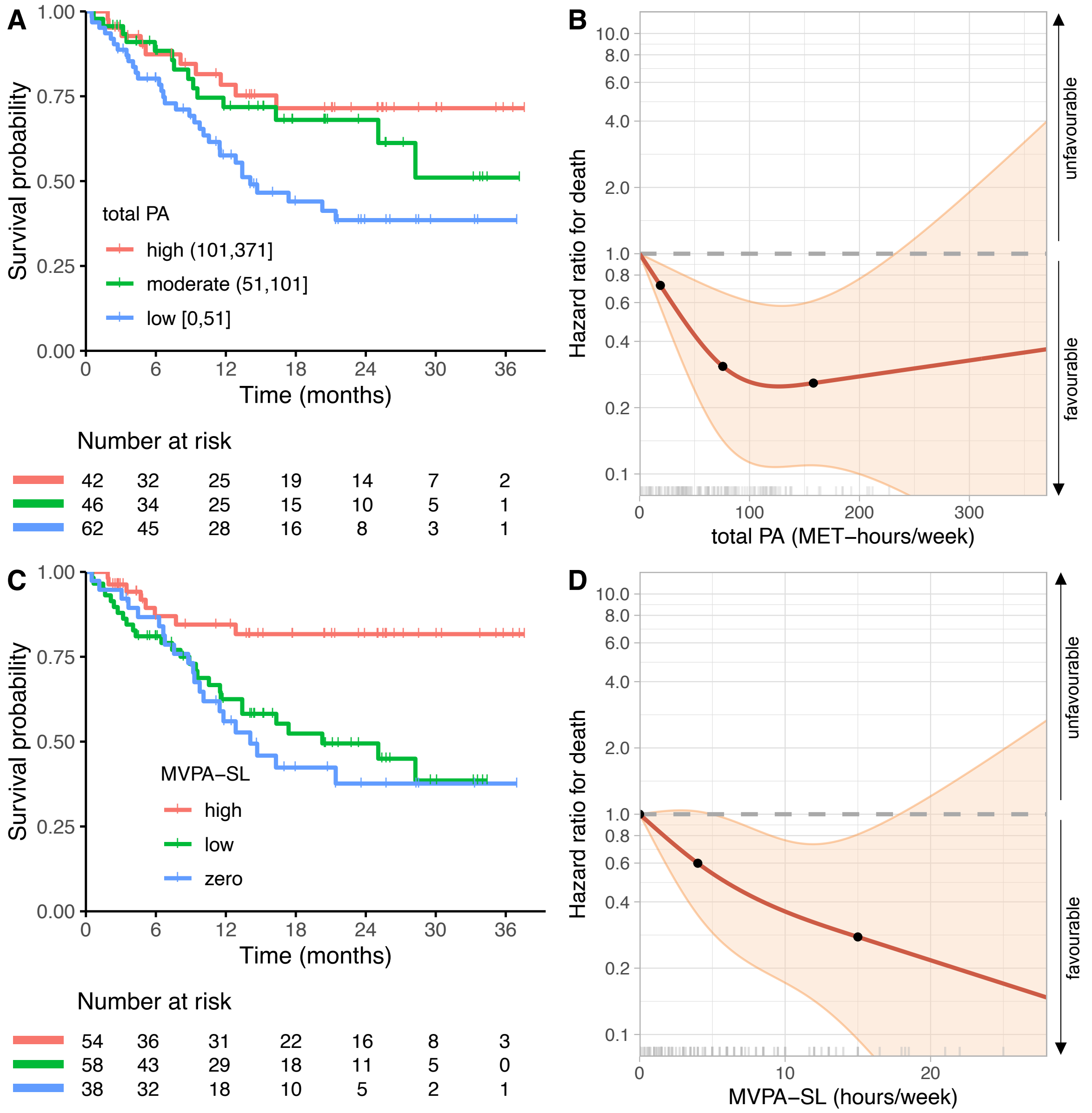

Supplementary Figure 6: Association between physical activity at immune checkpoint inhibitor initiation and overall survival in patients with metastatic or unresectable disease. Kaplan Meier curves (**a,c**) and restricted cubic spline models (**b,d**) of survival of patients with irresectable tumors treated with immune checkpoint inhibitors stratified by physical activity levels. **a,b** Total physical activity (Metabolic Equivalent Task (MET) hours per week). **c,d** Time spent on moderate-to-vigorous-intensity activities during sports and leisure time (MVPA-SL). Restricted cubic spline models (**b,d**) represent hazard ratio (HR) with 95% confidence interval (CI) with 0 as reference and are adjusted for sex, age, tumor type, previous systemic therapy, and type of therapy.

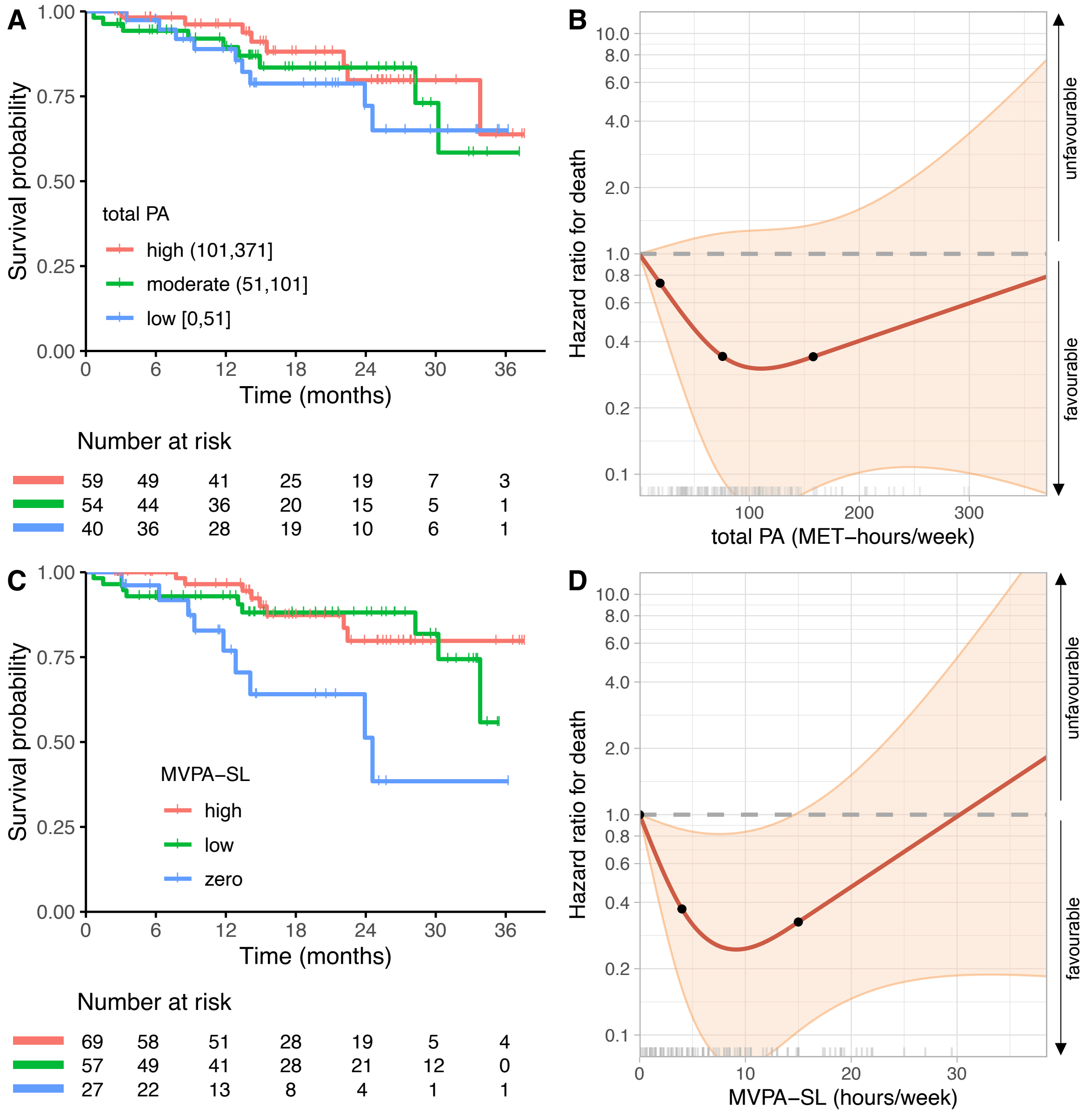

Supplementary Figure 7: Association between physical activity at immune checkpoint inhibitor initiation and overall survival in patients with melanoma. Kaplan Meier curves (**a,c**) and restricted cubic spline models (**b,d**) of survival of patients with melanoma treated with immune checkpoint inhibitors stratified by physical activity levels. **a,b** Metabolic Equivalent Task (MET) hours per week. **c,d** Time spent on moderate-to-vigorous-intensity activities during sports and leisure time (MVPA-SL). Restricted cubic spline models (**b,d**) represent hazard ratio (HR) with 95% confidence interval (CI) with 0 as reference and are adjusted for sex, age, treatment setting, previous systemic therapy, type of therapy, and lactate dehydrogenase (LDH).

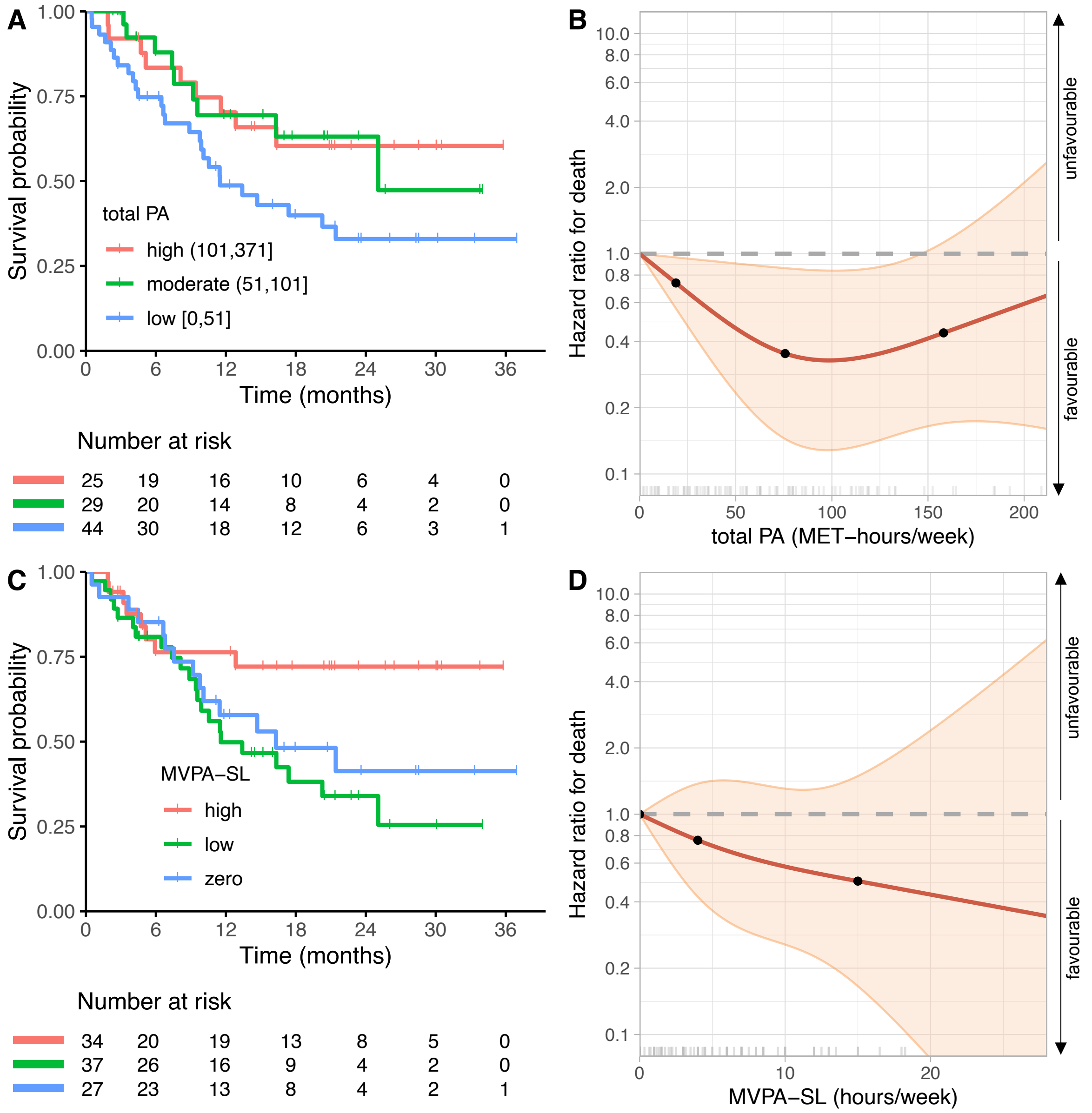

Supplementary Figure 8: Association between physical activity at immune checkpoint inhibitor initiation and overall survival in patients with nonmelanoma tumors. Kaplan Meier curves (**a,c**) and restricted cubic spline models (**b,d**) of survival of patients with melanoma treated with immune checkpoint inhibitors stratified by physical activity levels. **a,b** Metabolic Equivalent Task (MET) hours per week. **c,d** Time spent on moderate-to-vigorous-intensity activities during sports and leisure time (MVPA-SL). Restricted cubic spline models (**b,d**) represent hazard ratio (HR) with 95% confidence interval (CI) with 0 as reference and are adjusted for sex, age, treatment setting, previous systemic therapy, and type of therapy.

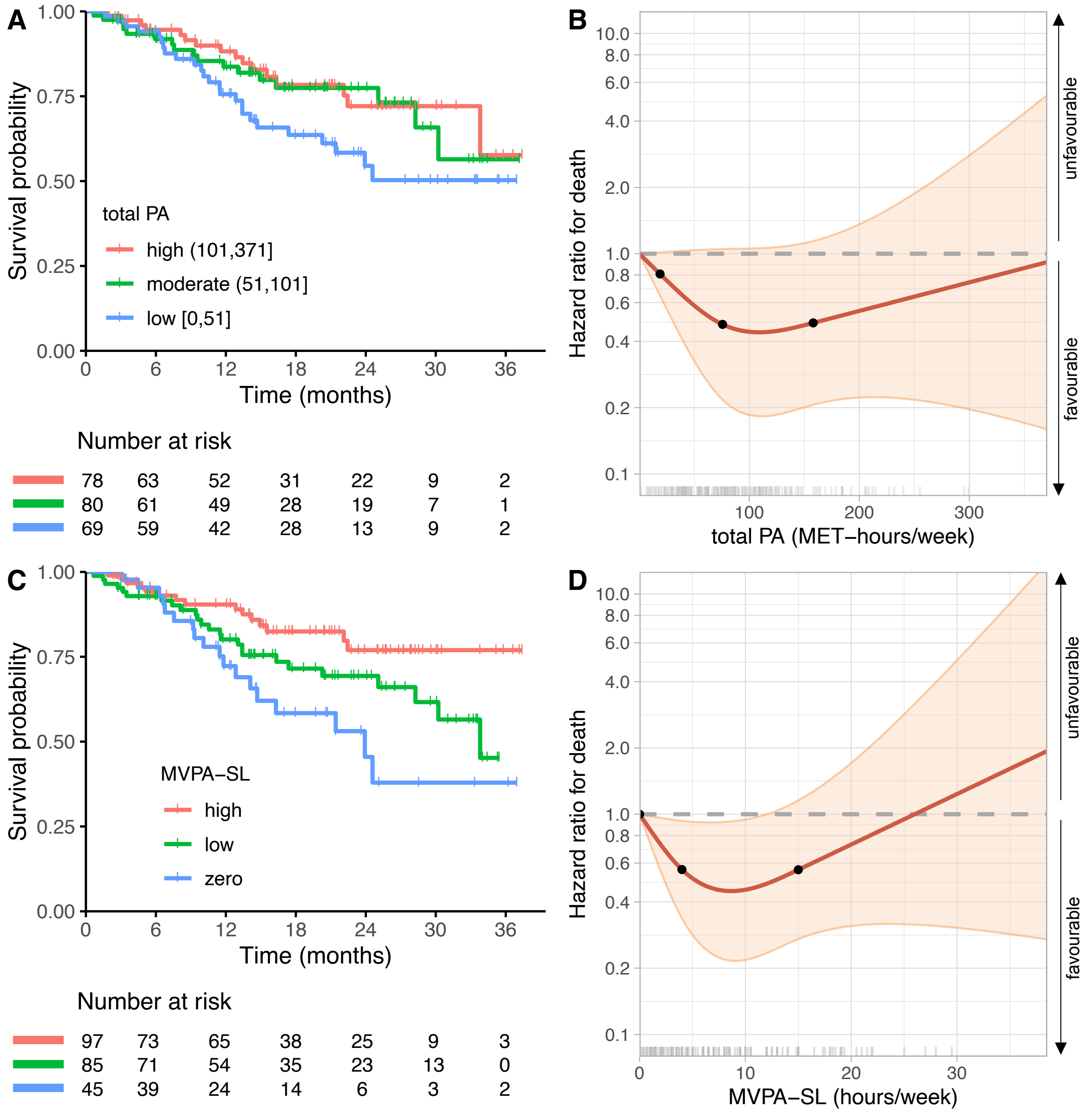

Supplementary Figure 9: Association between physical activity at immune checkpoint inhibitor initiation and overall survival in patients with cancer with ECOG performance status 0 or 1. Kaplan Meier curves (**a,c**) and restricted cubic spline models (**b,d**) of survival of patients with ECOG performance status 0 or 1 treated with immune checkpoint inhibitors stratified by physical activity levels. **a,b** Time spent on moderate-to-vigorous-intensity activities during sports and leisure time (MVPA-SL). **c,d** Metabolic Equivalent Task (MET) hours per week. Restricted cubic spline models (**b,d**) represent hazard ratio (HR) with 95% confidence interval (CI) with 0 as reference and are adjusted for sex, age, treatment setting, previous systemic therapy, and type of therapy.

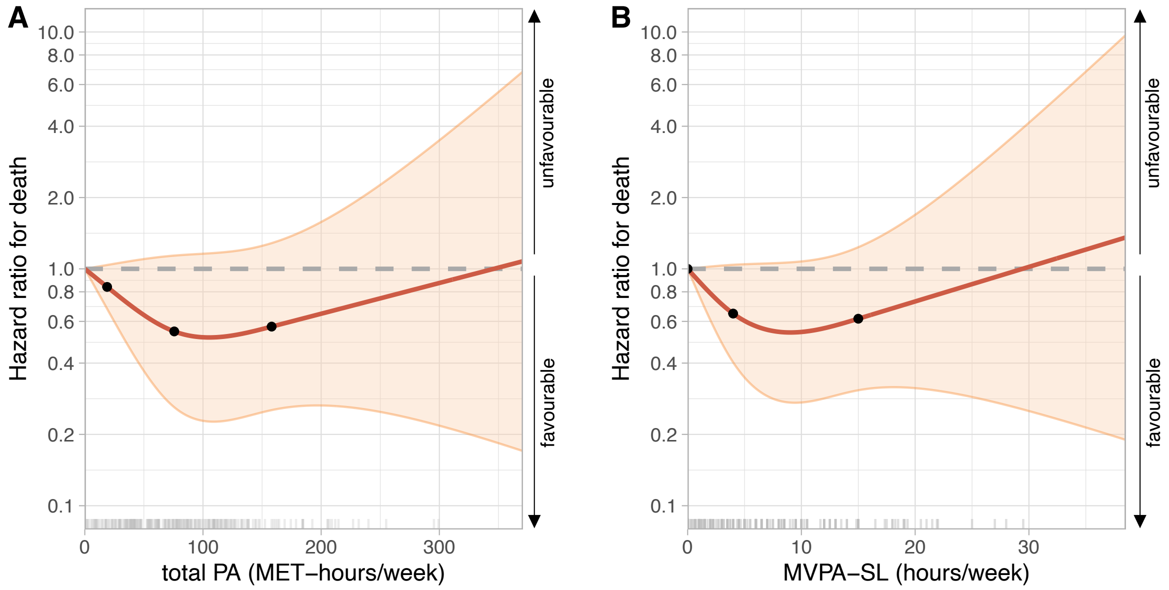

Supplementary Figure 10: Association between physical activity at immune checkpoint inhibitor initiation and overall survival (OS) in patients with cancer with additional adjustment for ECOG performance status. a Metabolic Equivalent Task (MET) hours per week. **b** Time spent on moderate-to-vigorous-intensity physical activity during sports and leisure time (MVPA-SL). These curves represent hazard ratio (HR) with 95% confidence interval (CI) with 0 as reference and are adjusted for sex, age, ECOG performance status, tumor type, treatment setting, previous systemic therapy and type of therapy.
